# A computational analysis of human rights discourse in news media using institutional theory

**DOI:** 10.3389/fdata.2026.1899143

**Published:** 2026-07-31

**Authors:** Joel Andrew B. Cruz Jr, Ma. Rowena R. Caguiat

**Affiliations:** Department of Information Technology, College of Computer Studies, De La Salle University, Manila, Philippines

**Keywords:** computational discourse analysis, data-driven storytelling, human rights journalism, institutional theory, media framing

## Abstract

**Introduction:**

News coverage of politically sensitive events involves framing choices that accumulate across thousands of headlines, years, and media institutions. Likewise, no single reader or journalist can track these patterns as they happen. This study examines how English-language news media framed Rodrigo Duterte's anti-drug campaign in the Philippines from June 2016 to June 2025.

**Methods:**

The study applies BERTopic topic modeling and VADER sentiment analysis to 1,133 headlines collected through Google News's RSS endpoint. Institutional Theory organizes the results around three pressures: normative, coercive, and mimetic.

**Results:**

Ten topic clusters emerged from the corpus, centered on human rights violations, International Criminal Court (ICC) legal proceedings, and post-presidency accountability narratives. Domestic Philippine outlets covered human rights themes in a larger share of headlines than international outlets during the campaign's early years, but that pattern reversed after Duterte left office in 2022. By 2024 and 2025, international outlets covered human rights themes in roughly 64 percent of headlines, compared with about half for domestic outlets. Sentiment analysis classified 807 headlines as negative, 203 as neutral, and 123 as positive. Negative coverage rose sharply in early 2025, around Duterte's arrest on an ICC warrant. Headline language also clustered around three institutional events: the 2018 ICC filing, the 2020 United Nations (UN) Human Rights Report, and the 2021 ICC investigation authorization, each marked by distinct word patterns across positive, negative, and neutral coverage.

**Discussion:**

Normative pressure explains why domestic outlets led on human rights framing before any international body had acted. Coercive pressure from the ICC and UN corresponds to the sentiment spikes around each event. Mimetic pressure explains how framing conventions moved between domestic and international outlets over time, first from domestic to international sources and later in the other direction. The study also considers how Google News's own curation choices shape which outlets and frames appear in the corpus. These findings suggest computational analysis is both a research method and a way to hold a media record open to scrutiny after the news cycle moves on.

## 1 Introduction

Rodrigo Duterte's presidency in the Philippines lasted from 2016 to 2022 and brought one of the most intensively scrutinized law enforcement campaigns in recent Southeast Asian history. His administration's war on drugs involved aggressive police operations that resulted in thousands of deaths. Human rights organizations disputed these as unlawful killings that targeted not only suspected drug dealers but also users and civilians with no established involvement in the drug trade (Human Rights Watch, 2025). The campaign reshaped the Philippine criminal justice system and deepened existing inequalities in marginalized communities (Barera, 2019). While Duterte had domestic support for his stance on crime prevention, some organizations raised formal concerns. Institutions such as the United Nations and the International Criminal Court voiced out due process violations that drew out global media attention (Bequelin, 2019; Xu and Geshman, 2016)

Media coverage of these events did more than document them. News headlines carry weight in shaping how audiences interpret political violence because they compress editorial judgment into a few words (Lamchek and Jopson, 2024; Morstatter et al., 2018). However, framing choices are not straightforward. What drives these framing choices, however, is not straightforward. Editorial decisions reflect organizational norms, professional standards, and the constraints imposed by digital platforms like Google News (Galeazzi et al., 2023). As an algorithmic aggregator, Google News does not produce original content but shapes narrative visibility by ranking, clustering, and filtering headlines from thousands of sources (Trielli and Diakopoulos, 2019). Its selection logic weighs relevance, recency, and source credibility in ways that determine which frames reach public attention (Petre, 2021; Ulloa and Kacperski, 2024). Therefore, Google News can also be treated as an institutional actor, and not merely a conduit for news stories.

What prior computational work on the drug war has largely left unaddressed is how these platform-level decisions interact with the framing choices of individual outlets over time. Studies engaging with the campaign's human rights dimensions have been extensive, but the dominant approach relies on qualitative methods or case studies limited to particular outlets or time windows (Geçer and Mahinay, 2018; Lamchek and Jopson, 2024). These approaches yield depth but are not well designed to track how framing patterns shift across years and across different media institutions at once (Degen et al., 2024). This study applies computational methods, specifically BERTopic topic modeling and VADER sentiment analysis, to headlines retrieved via Google News's RSS search endpoint. The analysis covers how domestic and international media framed Duterte's drug war from June 2016 to June 2025 and how that framing responded to major institutional events such as ICC filings and UN Human Rights Council reports.

Coercive, normative, and mimetic pressures, as described in Institutional Theory, help explain why media coverage of politically sensitive events tends to converge across outlets and why that coverage can shift when authoritative external actors intervene (Benson, 2004; Maniou, 2023). Applying this framework to a computational dataset makes the connection between broader institutional dynamics and observable shifts in headline language and sentiment concrete and traceable.

Three research questions guide this study: (1) *How Duterte's drug war was framed across the coverage period*; (2) *To what extent human rights themes appeared in that coverage*; and (3) *How major institutional events influenced headline tone and framing*. The corpus consists of English-language headlines retrieved through Boolean search queries. The analysis focuses on headlines rather than full articles because they provide a consistent, comparable unit of framing across sources and time. The timeframe extends beyond Duterte's presidency to capture how narratives persisted or shifted under the Marcos Jr. administration.

## 2 Literature review

### 2.1 Media framing of political violence and human rights

Framing shapes how audiences make sense of political events, not by distorting facts but by selecting which aspects of a story receive emphasis (Entman, 1993). In coverage of state violence, this selection process carries real consequences. Coverage of Duterte's drug war reflected this tension directly. Lamchek and Jopson (2024) Domestic Philippine outlets tended to frame police operations as a necessary public safety measure, while international outlets more consistently centered the deaths, the absence of due process, and the accounts of victims' families (Lamchek and Jopson, 2024; Reyes, 2016). These differences did not stem from one side having better information than the other. They reflect the distinct editorial cultures, audience expectations, and institutional pressures that each type of outlet operates under (Hosseinmardi et al., 2025). Understanding why coverage diverged and sometimes converged requires a framework that accounts for those pressures, which is where Institutional Theory becomes useful.

### 2.2 Institutional theory

Institutional Theory starts with a simple observation: organizations do not make decisions solely based on what is most efficient or logical. They also respond to the expectations of their environment, adjusting their behavior to appear credible and legitimate within it (Benson, 2004). Dimaggio and Powell (1983) described three ways this happens. The first is coercive pressure, where governments, regulators, or powerful institutions set boundaries that organizations feel compelled to follow. The second is normative pressure, where shared professional values do the same work more quietly. Journalists across different newsrooms, for instance, tend to follow similar ethical standards about fairness and accountability regardless of where they work (Oso et al., 2024). The third is mimetic pressure, where organizations follow the lead of others they respect, especially when they are unsure how to handle a difficult situation. For this study, these three pressures offer a way to read the patterns in media coverage without treating them as random. The framework does not explain everything, but it provides a grounded way to ask why coverage looked the way it did at different points in time (Maniou, 2023).

## 3 Methods

**Figure 1:**
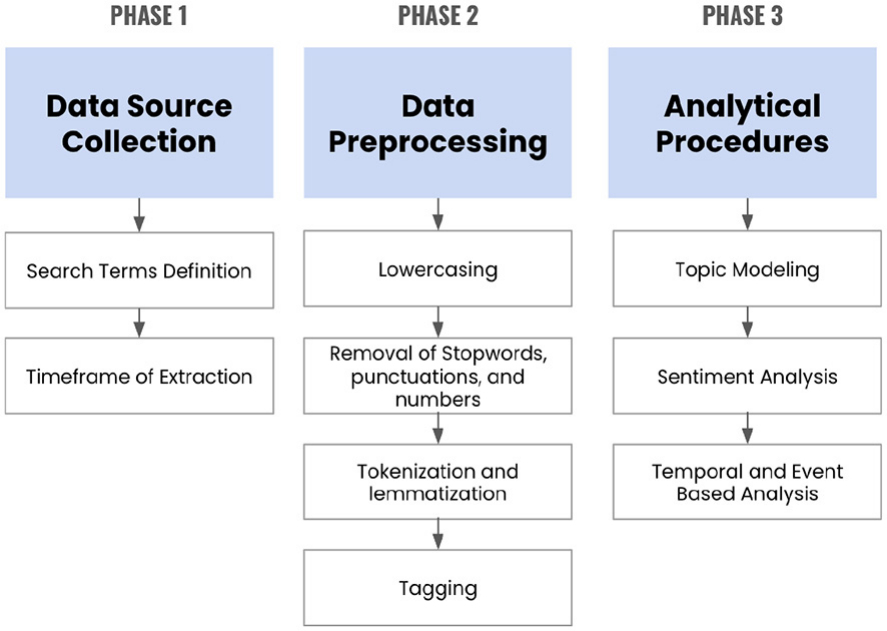
Research methodology.

This study investigates how news media framed Rodrigo Duterte's anti-drug campaign, with particular attention to human rights narratives. The design follows three sequential phases: data collection, data preprocessing, and computational analysis (as seen in Figure 1). Institutional Theory guides the interpretation throughout, with coercive, normative, and mimetic pressures serving as the framework for reading patterns in headline language and sentiment across time.

### 3.1 Data collection

Python was the primary programming language for all data collection, preprocessing, and analysis tasks, chosen for its range of natural language processing libraries. All scripts were written and executed in Google Colab, a cloud-based notebook environment.

Data collection began by defining 24 Boolean search queries across four thematic categories: war on drugs, extrajudicial killings, ICC investigation, and human rights (the full query set appears in Appendix A). These queries were designed to capture coverage from both domestic and international outlets across the full range of the campaign's public discourse. Each query was submitted to the Google News RSS endpoint using the parameters *hl*=*en-PH, gl*=*PH, and ceid*=*PH:en*, which set the language and regional context to English-language content from the Philippines. The scraper retrieved up to 100 results per query and introduced a one-second delay between requests to avoid server blocking. The coverage period ran from June 2016 to June 2025, spanning Duterte's full presidential term and extending into the post-presidency period to capture ongoing institutional and legacy narratives. After pooling results across all queries, duplicate headlines were removed using exact-match deduplication.

### 3.2 Data preprocessing

Raw headlines were cleaned before analysis. All text was lowercased to remove case distinctions, and punctuation, stopwords, and numbers were stripped to focus the analysis on content-bearing terms. The cleaned text was then tokenized into individual word units, and each token was lemmatized to its base dictionary form, so that variants such as “*killed”* and “*killing”* were treated as the same term. Each headline was also tagged with metadata including its publication date, outlet category (domestic or international), and any association with a pre-defined institutional event window.

Domestic outlets were defined as Philippine Daily Inquirer, Rappler, Manila Bulletin, Philippine Star, CNN Philippines, ABS-CBN News, and GMA News. All other sources in the dataset were classified as international. Afterwards, VADER sentiment analysis was applied to the original, unprocessed headlines rather than the cleaned versions, because VADER relies on capitalization, punctuation, and negation markers.

### 3.3 Analytical procedures

#### 3.3.1 Topic modeling

**Figure 2:**
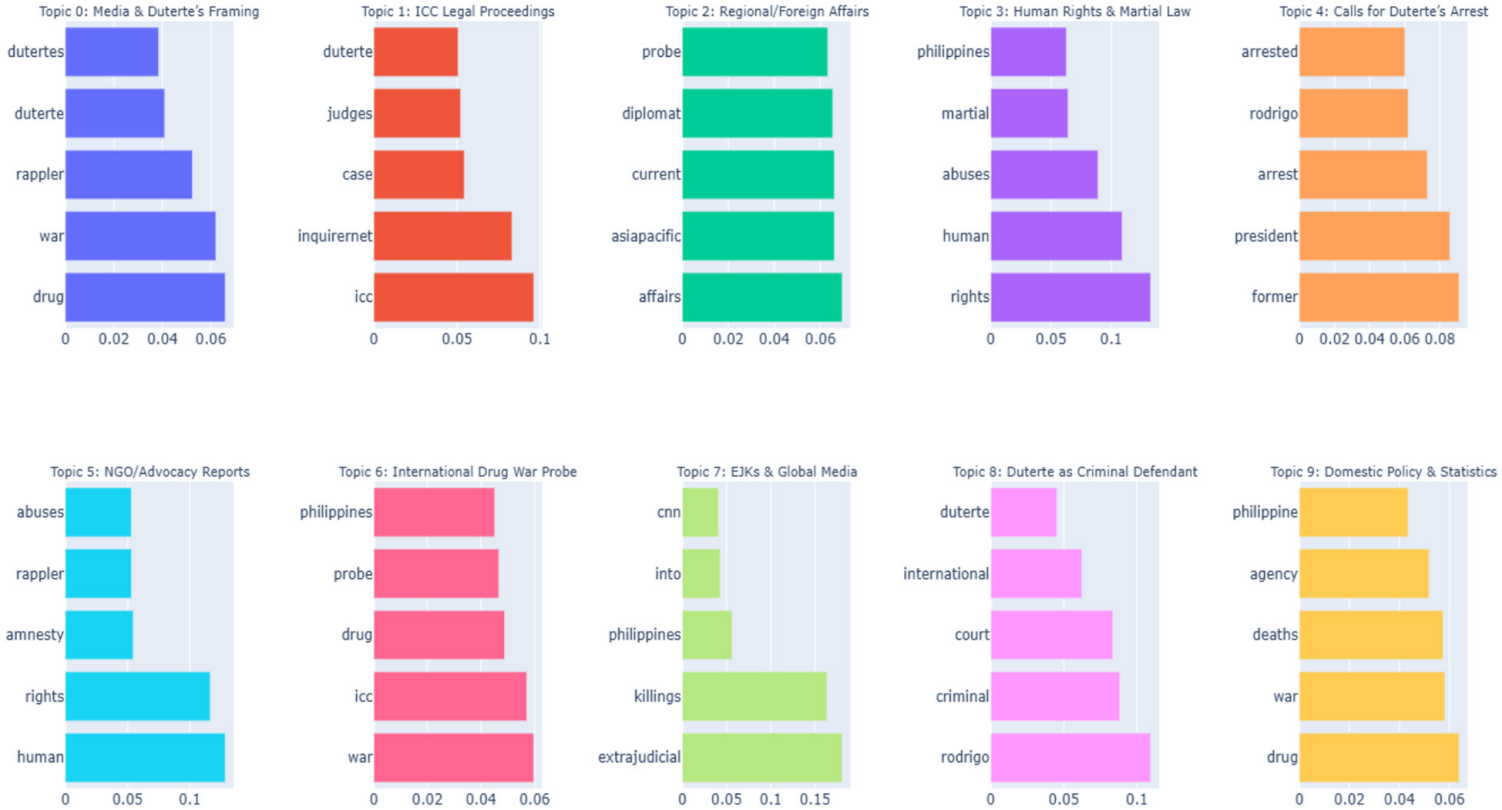
BERTopic clusters.

Topic modeling was carried out using BERTopic, an unsupervised machine learning approach that uses sentence-level transformer embeddings to group semantically similar texts into clusters. BERTopic was run with default parameters, using the all-MiniLM-L6-v2 sentence transformer, UMAP for dimensionality reduction, and HDBSCAN for clustering. The model produced ten topic clusters, each defined by its highest-scoring keywords. Thematic labels for each cluster were then assigned through manual interpretation of those keywords and are not auto-generated outputs of the model. Full UMAP and HDBSCAN parameter settings appear in Appendix B. Topic coherence was calculated using the c_v measure to assess how semantically consistent the words within each cluster are. Headlines that do not fit clearly into any of the 10 clusters are assigned to an outlier group and excluded from the labeled topics. BERTopic's default configuration does not fix a random seed for the UMAP dimensionality reduction step, so topic count and boundaries can vary slightly between runs on the same corpus. The topics reported in this study reflect one execution of the pipeline documented in Appendix B. Lastly, a second coder independently classified a random sample of 50 headlines into the 10 topic clusters, using the same keyword-based cluster definitions shown in Figure 2, without seeing the original classifications. Agreement between the two sets of labels was assessed using Cohen's kappa.

#### 3.3.2 Sentiment analysis

Sentiment scoring was performed using the Valence Aware Dictionary and sEntiment Reasoner (VADER), a lexicon-based tool designed for short, social-media-style text. Each headline received a compound score and was classified as positive (score ≥0.05), negative (score ≤ −0.05), or neutral (score between −0.05 and 0.05). VADER was chosen because it handles informal language and news-style phrasing without requiring model training. Afterwards, a random sample of 100 headlines was set aside for manual sentiment coding. A coder assigned each headline a positive, negative, or neutral label without reference to VADER's output, and agreement between the manual labels and VADER's classifications was assessed using Cohen's kappa.

#### 3.3.3 Time and event base analysis

To track how sentiment shifted in response to institutional events, three key dates were defined: (1) the ICC filing on February 8, 2018; (2) the UN Human Rights Report on June 4, 2020; and (3) the ICC investigation authorization on September 15, 2021. The 7 day window balances two competing needs: enough time to capture coverage that responds to an announcement with a short lag, and a window narrow enough to avoid absorbing unrelated news cycles from adjacent weeks. A shorter window risked missing follow-up coverage published a few days after each announcement, and a longer window risked mixing in coverage driven by unrelated developments. Headlines published within this window around each date were flagged and analyzed separately. Sentiment distributions within these windows were compared against baseline patterns to assess whether institutional events coincided with measurable changes in media tone. Word clouds were generated for each event and sentiment category to identify the specific language patterns that characterized each moment in the coverage.

## 4 Results

The following results are drawn from topic modeling, sentiment analysis, and event-based discourse analysis of 1,133 unique headlines collected from June 2016 to June 2025.

### 4.1 Core themes in news coverage

BERTopic identified 10 clusters in the headline corpus, each representing a distinct strand of media discourse around the drug war (See Table 1). Topic coherence for this run measured 0.468 using the c_v metric. A total of 276 headlines (24.4%) of the corpus, fell into the outlier group and are excluded from the 10 labeled topics discussed below. A second coder independently classified a random sample of 50 headlines into the 10 clusters using the same keyword lists shown in Appendix A. The two sets of labels matched on 41 of the 50 headlines, an observed agreement of 82 percent, with a Cohen's kappa of 0.80.

**Table 1:** Topic modeling results.

Topic	Top Keywords	Theme	Implications
**0**	drug, war, rappler, duterte, dutertes	Media and Duterte's Framing	Focus on **media** coverage and Duterte's narrative on the drug war
**1**	icc, inquirer, case, judges, duterte	ICC Legal Proceedings	Emphasizes **international legal scrutiny** via ICC and Inquirer's reporting.
**2**	affairs, asiapacific, current, diplomat, probe	Regional/Foreign Affairs	Coverage by foreign diplomats or ASEAN angle on the war on drugs.
**3**	rights, human, abuses, martial, philippines	Human Rights and Martial Law	Highlights **abuses** under the anti-drug campaign and allusions to **martial law**—rights violations central.
**4**	former, president, arrest, rodrigo, arrested	Calls for Duterte's Arrest	Suggests content around **post-presidency legal action** or advocacy for his arrest. Growing accountability discourse.
**5**	human, rights, amnesty, rappler, abuses	NGO/Advocacy Reports	NGOs (like Amnesty) and media (Rappler) documenting abuses.
**6**	war, icc, drug, probe, philippines	International Drug War Probe	A blend of domestic campaign and **external legal investigation**—likely broad framing of the crackdown.
**7**	extrajudicial, killings, philippines, into, cnn	EJKs and Global Media	Coverage by **CNN and others on EJKs**—most direct focus on violence and killings.
**8**	rodrigo, criminal, court, international, duterte	Duterte as Criminal Defendant	Legal framing of Duterte as a **defendant in international courts**—stigmatizing tone.
**9**	drug, war, deaths, agency, philippine	Domestic Policy and Statistics	Official reporting on **deaths, enforcement agencies**, and policy outcomes—possibly more neutral or state-driven.

The ten clusters reflect the main angles through which the drug war was covered over the study period. As seen in Figure 2, Topics 3, 5, and 7 center directly on human rights violations and extrajudicial killings, with Topic 7 showing the clearest link to international broadcast outlets such as CNN. Topics 1, 6, and 8 group around legal and institutional processes, particularly ICC proceedings. Topic 4, which contains keywords like “arrested” and “former president,” reflects post-presidency accountability narratives that emerged as Duterte's legal exposure grew after 2022. Topic 9 stands apart from the others by leaning toward more procedural, state-adjacent language around deaths and enforcement agencies, suggesting a more neutral or officially sourced framing.

### 4.2 Domestic vs. international coverage of human rights themes

**Figure 3:**
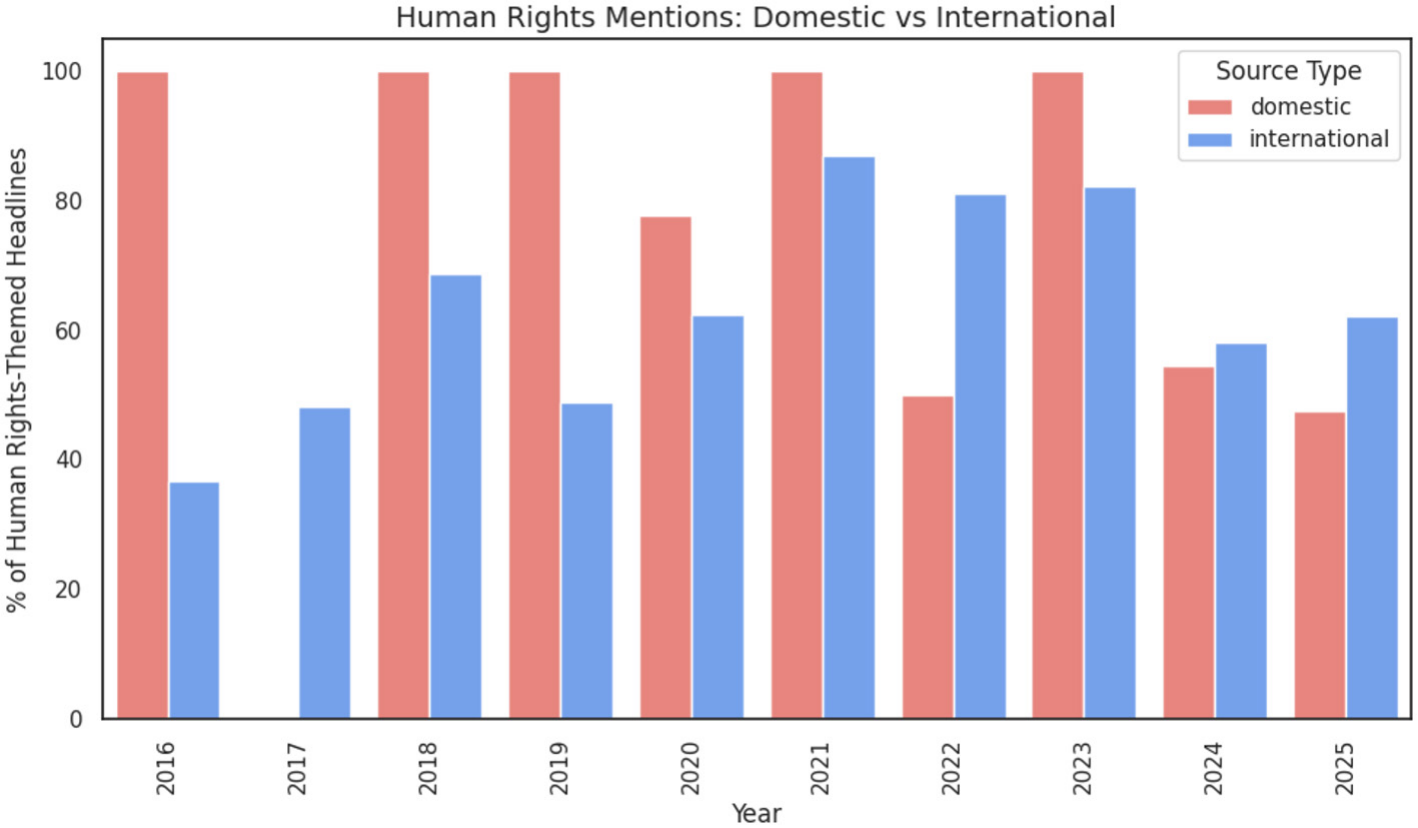
Percentage of human rights-themed headlines by year and outlet category (Domestic vs International News).

A comparison between domestic and international news sources shows how the roles of each shifted over the study period (See Figure 3). Full headline counts by year and outlet category appear in Appendix C. In the earliest years of the corpus, both outlet categories carry small headline counts, domestic ranging from five to twelve headlines a year and international from forty to seventy-six, so year-to-year percentages in this period move considerably. Domestic human rights framing reached 91.7% of headlines in 2018 but sat lower in 2016 and 2017, at 50.0 and 28.6%. International outlets covered human rights themes in a smaller share of headlines in these same 2 years, 34.9% and 50.0% percent, before rising alongside domestic coverage from 2018 onward.

From 2019 through 2023, both outlet categories maintained high shares of human rights-themed coverage. Domestic headlines reached peaked in 2021 and again in 2023, while international headlines ranged fluctuated across these years. Domestic coverage dropped to 70.0% in 2022, the year after Duterte left office, then surged in 2023, so a uniform post-2022 retreat is not evident at the year level within this window. Sample sizes across these years remain small, which limits how much weight any single year's percentage can carry.

The clearest divergence between outlet categories appears in 2024 and 2025, when headline counts grow large enough for the percentages to rest on a more stable base. Domestic coverage fell to 48.1% of headlines in 2024 and 55.4% in 2025. However, international coverage held higher over the same 2 years, at 64.6% and 63.7%. These 2 years carry the largest samples in the dataset and offer the most reliable basis for the finding that international outlets sustained human rights framing at a higher rate than domestic outlets did after Duterte's presidency ended. The Discussion section considers why domestic coverage receded over this period.

### 4.3 Sentiment distribution

**Figure 4:**
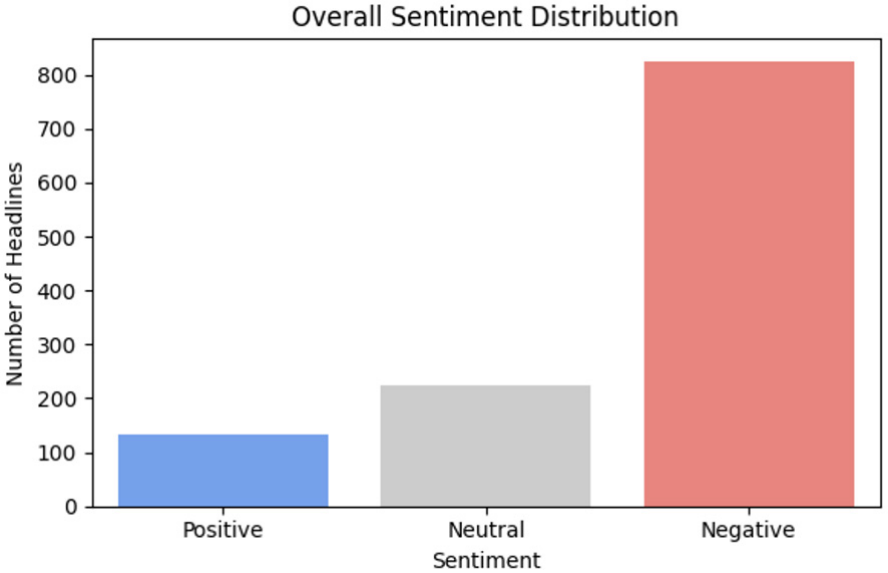
Sentiment analysis distribution.

Sentiment analysis showed that most headlines were classified as negative, with 807 headlines in this category (See Figure 4). Neutral headlines numbered 203, and 123 headlines were classified as positive. The dominance of negative sentiment reflects a consistently critical tone in coverage of the drug war across both domestic and international sources, driven largely by the subject matter itself: extrajudicial killings, impunity, and due process violations. Positive headlines were rare and, when they did appear, tended to come from coverage of institutional actions such as the ICC authorizing an investigation or the UN releasing a critical report, framed from the perspective of victims or human rights advocates rather than endorsements of the campaign.

Tracking sentiment over time reveals that negative coverage was relatively stable from 2016 through early 2023. In the first quarter of 2024, however, negative headlines rose sharply to over 260, far above any comparable period in the dataset. This surge coincides with intensified ICC activity and growing international legal pressure on Duterte, though no formal event window was defined for this period in the current analysis, and it warrants closer examination in future work. Lastly, a manual check of 100 randomly sampled headlines against VADER's classifications showed agreement on 86 of the 100, a Cohen's kappa of 0.71. See Table 2.

**Table 2:** VADER validation.

Sentiment	Positive	Neutral	Negative
Positive	8	1	11
Neutral	1	13	9
Negative	1	2	54
**Total**	**10**	**16**	**74**

### 4.4 Language patterns around institutional events

**Figure 5:**
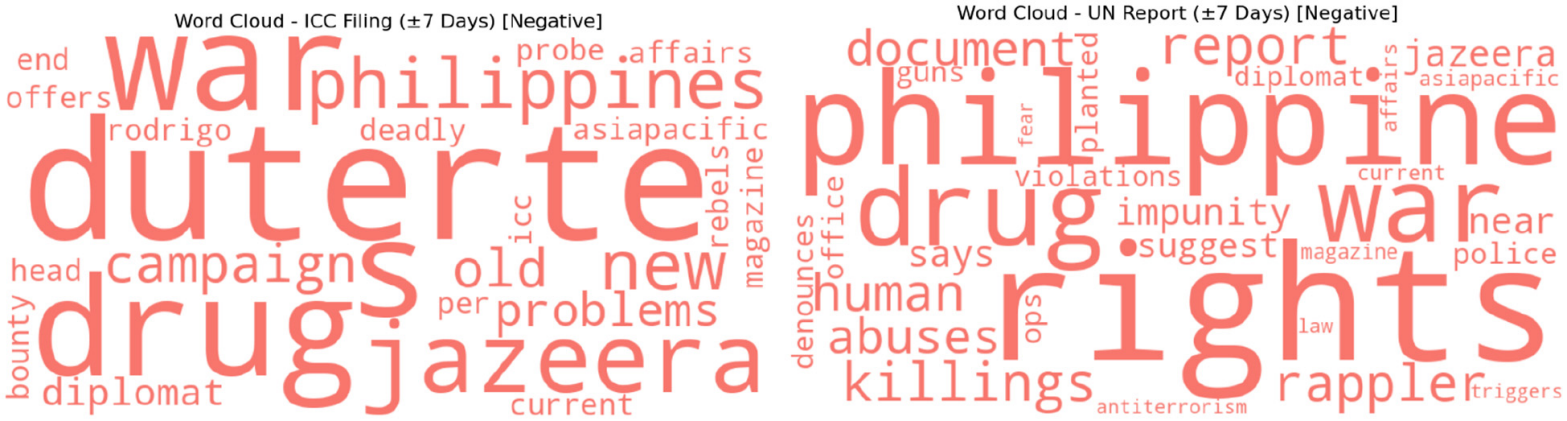
Word cloud (Negative).

Word clouds generated within the ±7-day windows around the three institutional events show distinct language patterns by sentiment category. For the ICC investigation window, negative headlines (See Figure 5) clustered around terms such as *war, killings, impunity, and police*. These terms tie the event to ongoing state violence rather than to the legal proceeding itself.

**Figure 6:**
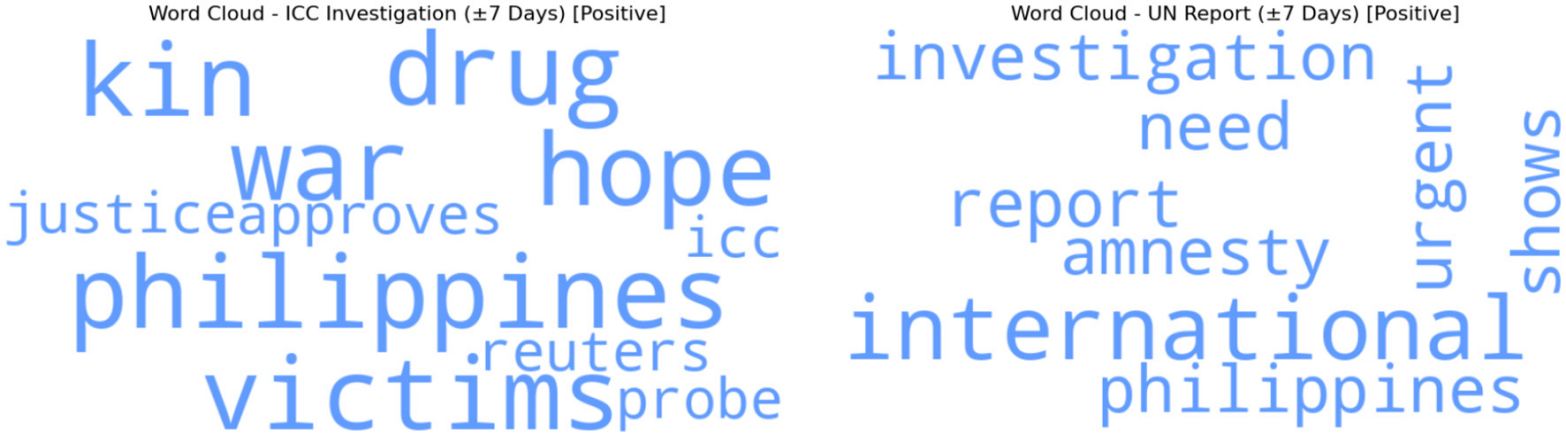
Word cloud (Positive).

**Figure 7:**
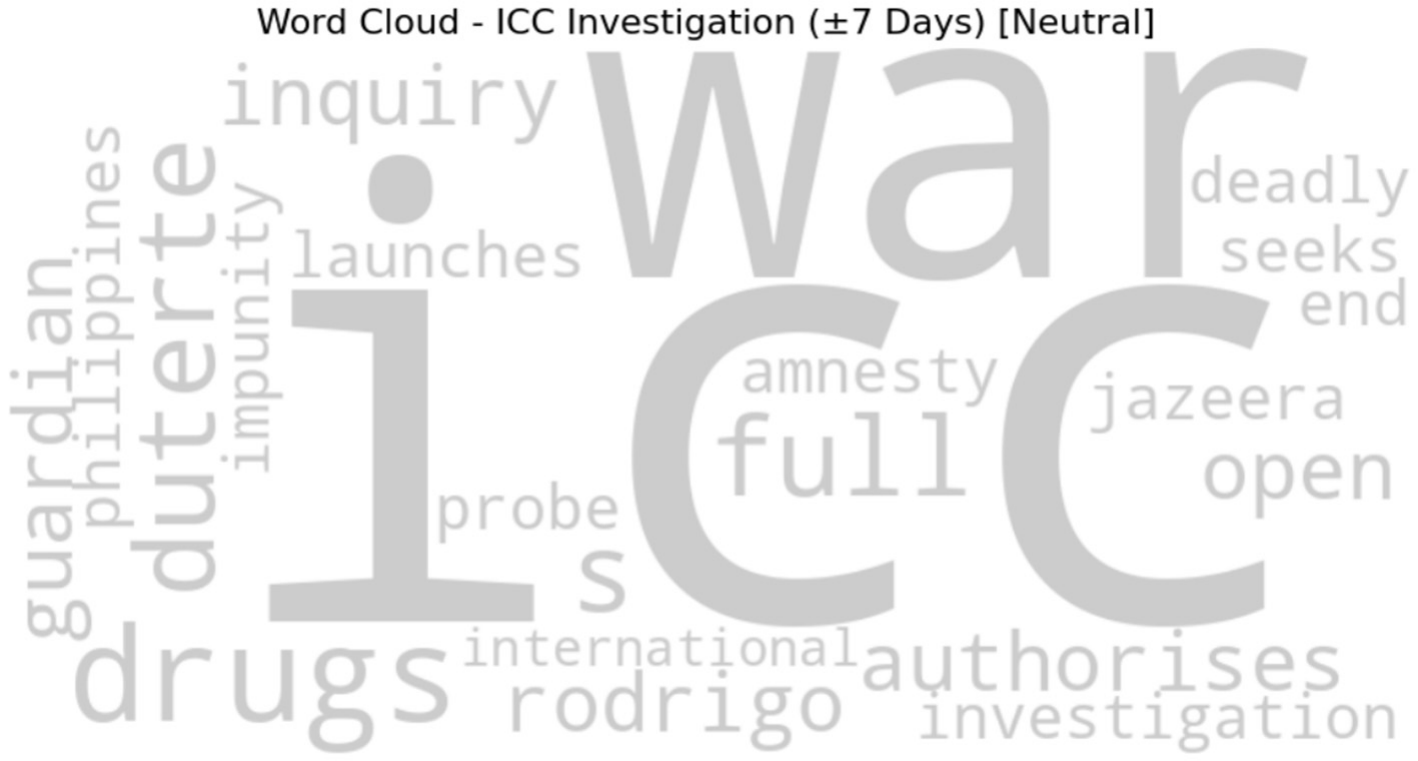
Word cloud (Neutral).

Positive headlines (See Figure 6) within the same window used terms such as hope, victims, justice, and kin, reflecting coverage oriented toward accountability and victim recognition. Neutral headlines (See Figure 7) used procedural language such as *authorizes, probe*, and *launches*, consistent with straight news reporting on institutional actions.

The UN Human Rights Report window produced similar patterns. Negative coverage used terms like killings, abuses, impunity, and planted, framing the state as the primary source of harm. Positive coverage stressed calls for reform through words such as investigation, international, and amnesty. Neutral headlines centered on state responses, with terms like *refuses, implement*, and *recommendations* pointing to the government's resistance to external recommendations.

## 5 Discussion

The results across topic modeling, sentiment analysis, and event-based analysis converge on a consistent pattern: media coverage of Duterte's drug war was shaped not only by the events themselves but by the institutional context in which those events occurred. Reading these patterns through Institutional Theory reveals how coercive, normative, and mimetic pressures each played a role in determining what got covered, when, and in what tone.

### 5.1 Institutional pressures and media behavior

Coercive pressure accounts most directly for the sentiment shifts around the three institutional events and the sharp rise in negative headlines during the first quarter of 2025. When the ICC moved from preliminary examination to formal investigation and then to an arrest warrant, media attention intensified. Formal legal authority from a recognized international body creates conditions in which organizations, including news outlets, also feel compelled to respond (Benson, 2004). The clearest instance of this came in March 2025, when Duterte was arrested on the ICC warrant and flown to The Hague. Negative headlines that quarter reached 266, the highest quarterly count in the dataset, driven by coverage of the arrest itself alongside disbarment complaints filed against him in the preceding weeks. The ICC's involvement did not only generate more coverage. It shifted the narrative toward accountability framing that had been present earlier but subdued, a pattern visible in the word clouds for each event window (See Figures 5 through 7).

Normative pressure appears earliest, in the behavior of domestic outlets before international legal pressure existed. Rappler and the Philippine Daily Inquirer produced human rights-centered coverage from the campaign's first months, drawing on professional journalism norms that treat holding power to account as a baseline obligation (Maniou, 2023). This coverage predates the ICC filing by nearly 2 years. The dataset therefore points to domestic professional norms, not external legal pressure, as a driver of critical framing in this study.

Lastly, mimetic pressure appears in how framing conventions moved between outlet types over the study period. In the early years, international outlets largely followed frames that domestic Philippine media had already established, picking up narratives of state violence and rights violations that originated in local reporting. In later years, the direction reversed. International outlets sustained human rights coverage that domestic sources had pulled back from. The language in Topic 7 shows international broadcast outlets carrying forward the same accountability vocabulary that Rappler and the Inquirer had used earlier. This movement between institutional environments matches how mimetic behavior operates under uncertainty, where organizations look to credible peers when a story lacks a clear domestic playbook (Benson, 2004; Maniou, 2023).

The three pressures explain different parts of the same pattern rather than compete as alternative explanations. Normative pressure opened the coverage, since domestic outlets applied professional watchdog norms before any international body had acted. Coercive pressure then produced the sharp, dated spikes in negative sentiment, each tied to a specific ICC or UN action. Mimetic pressure explains how framing moved between outlet types over time. None of the three pressures alone explains why domestic coverage declined while international coverage held steady after 2022. That shift required both the loss of the domestic news hook described above and the mimetic transfer of the accountability frame to outlets outside the domestic market. Coercive events, normative practice, and mimetic exchange together account for when the sentiment moved, who covered the story first, and who kept covering it once domestic conditions changed.

### 5.2 The role of google as an institutional actor

The dataset itself reflects Google News's role as more than a passive aggregator. Its RSS endpoint surfaces headlines according to an algorithmic logic that weighs recency, source authority, and topical clustering, meaning that the corpus collected here is already shaped by platform-level curation decisions (Trielli and Diakopoulos, 2019; Ulloa and Kacperski, 2024). The presence of certain outlets, particularly Rappler and the Philippine Daily Inquirer among domestic sources, and CNN and Al Jazeera among international ones, reflects not only editorial choices but also which sources Google News's algorithm treats as authoritative within this topic cluster.

The corpus reflects specific choices built into the retrieval process, not a general aggregation logic applied uniformly across queries. Each query in this study used the parameters which set language and regional context to English-language content from the Philippines. Appendix A lists the full query set. However, Google News has limited documentation on how its news ranking works. Google do not disclose publicly how the news platform combines relevance, recency, and weighting. Therefore, this leaves an open boundary between what this study can attribute to editorial choices at the outlet level and what belongs to platform-level curation.

The regional search parameters used for data collection mean that the corpus comes from the sources Google News ranks as relevant to a Philippines-based query. It does not come from a neutral global sample. International outlets that rank lower for a Philippines-based search, or that Google's algorithm considers less relevant locally, are likely underrepresented compared to their actual coverage volume. If this bias exists, the true difference between domestic and international coverage after 2022 is likely as large as what this study reports. The corpus likely misses some international coverage instead of inflating it. In contrast, domestic outlets are captured nearly fully within this topic area because the query parameters favor this regional context. The comparison between domestic and international coverage should be viewed as a conservative estimate of the difference between outlet types, rather than an overstatement.

### 5.3 Practical implications

The decline in domestic human rights coverage after 2022 carries practical implications for how accountability journalism survives political transitions. When local outlets reduce their critical coverage, the burden of sustaining that scrutiny falls on international media and civil society organizations. Journalists can counter issue fatigue by building dedicated accountability structures, such as editorial desks focused on transitional justice, rather than relying on sporadic event-driven surges (Dukalskis et al., 2024). Data-driven storytelling tools, including the kind of computational analysis used in this study, can help reporters identify patterns in institutional responses and track developments that might otherwise be missed in the day-to-day news cycle (Silva et al., 2023).

For policymakers, the consistency of negative sentiment across the study period, and its concentration around institutional events, points to the reputational costs of resisting international accountability mechanisms. Cooperation with ICC proceedings, independent monitoring bodies, and transparent reporting on enforcement outcomes could begin to shift the narrative over time (Dagher, 2018; Tiscornia, 2024). For international institutions, the findings suggest that major announcements carry greater media impact when paired with clear communication strategies. When ICC and UN actions are accompanied by victim-centered narratives and accessible factual context, coverage tends to incorporate more hopeful and justice-oriented language, as the word cloud analysis of positive sentiment windows shows (Apriliyanti et al., 2022; Shea et al., 2022).

## 6 Conclusion

This study shows that media coverage of Duterte's drug war was never just a record of what happened. Through their framing choices, news outlets actively shaped how the public understood the campaign, which voices were heard, and whether state violence was treated as policy or as wrongdoing. The computational analysis traced how those choices shifted across nearly a decade, how institutional events from the ICC and the UN periodically reset the tone of coverage, and how Google News quietly determined which of those stories found their way to audiences in the first place. What emerges from the data is not simply a timeline of headlines but a picture of how media power and institutional accountability interact over time.

The drug war may no longer dominate front pages, but its legacies have not faded. The cases before the ICC remain open, the families of victims are still waiting for answers, and the domestic outlets that once held the state accountable have grown quieter. What kept public memory of this period alive, and what continues to do so, is largely the work of international journalism and civil society. That asymmetry is itself a finding worth sitting with, because it raises a question that goes beyond the Philippines.

This is precisely where computational methods and data storytelling become more than research tools. The patterns uncovered in this study, from shifting sentiment to the language clusters that formed around moments of institutional intervention, were not visible to any single reader or journalist following the story in real time. They only became legible when the data was analyzed, interpreted, and communicated systematically. As AI continues to reshape how large datasets are processed and presented, studies like this one point to a broader opportunity: using intelligent, human-centered approaches to data communication not just to describe what the media did, but to hold that record open for scrutiny long after the news cycle has moved on. Transparency in how those tools work, and honesty about what they can and cannot reveal, remains as important as the insights they produce.

## Data Availability

The raw data supporting the conclusions of this article will be made available by the authors, without undue reservation.
