# Supplementary material for: A computational analysis of human rights discourse in news media using institutional theory
Source: Front Big Data. 2026 Jul 31;9:1899143. doi: 10.3389/fdata.2026.1899143 (PMC13472784; doi:10.3389/fdata.2026.1899143)
Supplement: Supplementary file 1 [file Data_Sheet_1.pdf]

Appendix A: Boolean Search Queries

Category	Search Queries
War on Drugs	"Rodrigo Duterte" AND "war on drugs"', "President Duterte" AND "drug war"', "Rodrigo Duterte" AND "anti-drug campaign", Duterte" AND "drug crackdown","Philippines" AND "Duterte" AND "drug war","Rodrigo Duterte" AND "drug-related killings"
Extrajudicial Killings	"Rodrigo Duterte" AND "extrajudicial killings"',"President Duterte" AND "summary executions","Duterte" AND "killings without trial","Philippines" AND "extrajudicial killings"', "Duterte government" AND "death squads","Rodrigo Duterte" AND "police abuse"
ICC Investigation	"Rodrigo Duterte" AND "ICC investigation”,"Duterte" AND "International Criminal Court","ICC" AND "Philippines drug war","Rodrigo Duterte" AND "crimes against humanity","Duterte" AND "Hague investigation","ICC" AND "extrajudicial killings Duterte"
Human Rights	“Rodrigo Duterte" AND "human rights violations", "Duterte" AND "UN human rights report", "Philippines" AND "rights abuses","Duterte" AND "Amnesty International","Rodrigo Duterte" AND "violence against civilians","Duterte" AND "human rights crisis"

Appendix B: BertTopic Parameters

Parameter	Detailed Description
Embedding Model	`all-MiniLM-L6-v2 (sentence-transformers)`
Uniform Manifold Approximation and Projection for Dimension Reduction (UMAP)	`n_neighbors = 15`<br>`n_components = 5`<br>`min_dist = 0.0`<br>`metric = cosine.`
Hierarchical Density-Based Spatial Clustering of Applications with Noise (HDBSCAN)	`min_cluster_size = 10`<br>`metric = euclidean,`<br>`cluster_selection_method = eom.`
Vectorizer	`CountVectorizer` with default English tokenization<br>`ngram_range = (1, 1).`
Others	No random seed was fixed for the UMAP step, so topic count and boundaries can vary slightly between runs on the same corpus

Appendix C: Headline Counts by Year and Outlet Category

Year	Domestic	International	Total
2016	6	43	49
2017	7	76	83
2018	12	40	52
2019	5	44	49
2020	12	45	57
2021	9	40	49
2022	10	47	57
2023	9	40	49
2024	79	113	192
2025	168	328	496
